# ColE1-Plasmid Production in *Escherichia coli*: Mathematical Simulation and Experimental Validation

**DOI:** 10.3389/fbioe.2015.00127

**Published:** 2015-09-01

**Authors:** Inga Freudenau, Petra Lutter, Ruth Baier, Martin Schleef, Hanna Bednarz, Alvaro R. Lara, Karsten Niehaus

**Affiliations:** ^1^Abteilung für Proteom- und Metabolomforschung, Fakultät für Biologie, Universität Bielefeld, Bielefeld, Germany; ^2^PlasmidFactory GmbH & Co. KG, Bielefeld, Germany; ^3^Institut für Genomforschung und Systembiologie, Centrum für Biotechnologie (CeBiTec), Universität Bielefeld, Bielefeld, Germany; ^4^Departamento de Procesos y Tecnología, Universidad Autónoma Metropolitana-Cuajimalpa, Mexico City, Mexico

**Keywords:** plasmid replication, small RNA, modeling, ordinary differential equations, uncharged tRNA, high copy plasmid, biotechnology

## Abstract

Plasmids have become very important as pharmaceutical gene vectors in the fields of gene therapy and genetic vaccination in the past years. In this study, we present a dynamic model to simulate the ColE1-like plasmid replication control, once for a DH5α-strain carrying a low copy plasmid (DH5α-pSUP 201-3) and once for a DH5α-strain carrying a high copy plasmid (DH5α-pCMV-lacZ) by using ordinary differential equations and the MATLAB software. The model includes the plasmid replication control by two regulatory RNA molecules (RNAI and RNAII) as well as the replication control by uncharged tRNA molecules. To validate the model, experimental data like RNAI- and RNAII concentration, plasmid copy number (PCN), and growth rate for three different time points in the exponential phase were determined. Depending on the sampled time point, the measured RNAI- and RNAII concentrations for DH5α-pSUP 201-3 reside between 6 ± 0.7 and 34 ± 7 RNAI molecules per cell and 0.44 ± 0.1 and 3 ± 0.9 RNAII molecules per cell. The determined PCNs averaged between 46 ± 26 and 48 ± 30 plasmids per cell. The experimentally determined data for DH5α-pCMV-lacZ reside between 345 ± 203 and 1086 ± 298 RNAI molecules per cell and 22 ± 2 and 75 ± 10 RNAII molecules per cell with an averaged PCN of 1514 ± 1301 and 5806 ± 4828 depending on the measured time point. As the model was shown to be consistent with the experimentally determined data, measured at three different time points within the growth of the same strain, we performed predictive simulations concerning the effect of uncharged tRNA molecules on the ColE1-like plasmid replication control. The hypothesis is that these tRNA molecules would have an enhancing effect on the plasmid production. The *in silico* analysis predicts that uncharged tRNA molecules would indeed increase the plasmid DNA production.

## Introduction

Since the finding that genetically engineered DNA can be used for gene therapy and DNA vaccination in the early 90s last century, the interest in plasmid DNA (pDNA) as a pharmaceutical gene vector has increased constantly (Kutzler and Weiner, 2008). The investigation of DNA vaccination in the past years shows enticing results in several areas, especially in prophylactic vaccine strategies and the usage of pDNA as potential therapeutics for the treatment of infectious diseases, cancer, Alzheimer disease, and allergies (Kutzler and Weiner, 2008; Mairhofer and Lara, 2014). A very promising field of application might be the genetic vaccination with DNA molecules to induce an immune response (Schleef and Blaesen, 2009). Using DNA molecules in that field has the advantage that there are no safety concerns associated with live vaccines. Additionally, the manufacturing process is short and stable in contrast to conventional vaccines (Kutzler and Weiner, 2008; Lara and Ramírez, 2012). It is expected that, since DNA vaccines and non-viral gene therapies enter phase 3 clinical trials and are approved for utilization, the demand for pDNA will increase (Bower and Prather, 2009). To meet these requirements, industrial-scale production processes of pDNA with adequate bacterial strains and vectors have to be designed. Therefore, it is important to understand which factors influence the plasmid replication.

In this work, we present a mathematical model to simulate the regulation of ColE1-like plasmid replication. Bacterial plasmids control their copy number through negative regulatory feedback mechanisms that adjust the replication rate (del Solar and Espinosa, 2000). In the case of ColE1-like plasmids, the replication is controlled by an antisense RNA molecule, which inhibits the maturation of the necessary primer RNA molecule for the DNA polymerase I (Grabherr and Bayer, 2002). When replication occurs, a part of an RNA preprimer transcript, named RNAII, binds to the plasmid origin of replication (oriV), where it forms a persistent hybrid (Itoh and Tomizawa, 1980). In the next step, it must be cleaved by the enzyme RNase H, which is specific for DNA–RNA hybrids (Schumann, 2001). This cleavage is essential to release the 3’OH group, so that the elongation by DNA polymerase I can start (Itoh and Tomizawa, 1980). To prevent the replication, this modification of RNAII by RNase H can be blocked through binding of a small complementary RNA transcript, named RNAI, which is the specific inhibitor of primer formation (Tomizawa and Itoh, 1981). RNAI and RNAII are coded within the same DNA region that is part of the oriV (Tomizawa, 1984). RNAI is constitutively transcribed from the opposite strand and regulates the frequency of replication initiation (Tomizawa, 1984). RNAI and RNAII form a transient, the so-called “kissing complex,” thus the RNase H cannot modify the RNA preprimer transcript anymore (Tomizawa, 1984, 1985). This complex is stabilized by the binding of the Rom protein (also called Rop protein) (Tomizawa, 1986, 1990). Additionally, the plasmid replication control can be affected by starvation conditions, which leads to a drop in free amino acids, and thus to an increase of uncharged tRNA molecules (Yavachev and Ivanov, 1988). If an uncharged tRNA molecule binds to an RNAI or RNAII molecule, no kissing complex can be built and the RNAII transcript can serve as a preprimer (Grabherr and Bayer, 2002). Consequently, a high amount of uncharged tRNA molecules is correlated with an increase in plasmid copy number (PCN) (Wróbel and Węgrzyn, 1998). Two models are proposed to describe the interaction between uncharged tRNA molecules and the regulatory RNA molecules. Yavachev and Ivanov (1988) found structural and sequence similarities between the loops of RNAI, respectively, RNAII and the cloverleaf structure of certain tRNA molecules. The second model was introduced by Wang et al. (2002), who suggest that the binding between an uncharged tRNA molecule and one of the regulatory RNA molecules takes place at the amino acid binding site. Although there are kinetic models, which explain the regulation of the ColE1-like plasmid replication (Ataai and Shuler, 1986; Bremer and Lin-Chao, 1986; Keasling and Palsson, 1989a,b; Brendel and Perelson, 1993; Wang et al., 2002), these models consider only parts of the ColE1-like plasmid replication control. Brendel and Perelson (1993), for instance, describe a model for the *in vivo* replication control mechanism by the regulatory RNA molecules, which includes kinetic information and accounts for measured concentration values.

In this study, the model proposed by Brendel and Perelson was extended. Their model describes the ColE1-like plasmid replication control by RNAI and RNAII molecules, with or without the interaction of Rom protein. They investigated plasmid concentrations at two different growth rates and showed a decrease of the PCN in presence of an intact *rom* gene as well as an increase of plasmid production at low growth rates (Brendel and Perelson, 1993). Apart from the replication control by the two regulatory RNA molecules, the plasmid replication can be influenced by uncharged tRNA molecules under amino acid starvation conditions (Wróbel and Węgrzyn, 1998). The mathematical model proposed in the present study incorporates the ColE1-like plasmid replication control by RNAI and RNAII molecules with or without the Rom protein. Additionally, the regulation by uncharged tRNA molecules is described. The advantage of this model is that it is confirmed by *in vitro* measurements of the plasmid concentration at different time points and the appropriate growth rates. Additionally, the free intracellular RNAI and RNAII concentrations were determined via quantitative reverse transcription real-time-PCR (qRT-PCR) for the same time points. With these data, the model was fitted and validated, so it could be used for *in silico* analysis of the ColE1-like plasmid replication control. Since this model takes into account the regulation by modified tRNA molecules, which cannot be charged by amino-acyl-tRNA synthetases anymore, it is possible to investigate the effect on the ColE1-like plasmid replication control.

## Materials and Methods

### Bacterial strain and plasmids

The *Escherichia coli* strain DH5α (F–Φ80*lac*ZΔM15 Δ(*lac*ZYA-*arg*F) U169 *rec*A1 *end*A1 *hsd*R17 (rK−, mK+) *pho*A *sup*E44 λ− *thi*-1 *gyr*A96 *rel*A1) (Source: Plasmid Factory, Bielefeld, Germany) was used as a host strain for transformation of the high copy plasmid pCMV-lacZ (Source: Plasmid Factory, Bielefeld, Germany) as well as the low copy plasmid pSUP 201-3 (Simon et al., 1983). pCMV-lacZ is a ColE1-derived high copy plasmid for therapeutic pDNA production with a size of 7164 bp. It contains a pBR322 origin of replication without the *rom* gene and carries genetic elements for DNA vaccination together with the ampicillin-resistance gene *bla*. The low copy plasmid pSUP 201-3 is also a ColE1-derivative and has a size of 7896 bp. It contains a pBR322 origin of replication together with the *rom* gene and carries a specific recognition site for mobilization. For selection, the ampicillin-resistance gene *bla* and a chloramphenicol resistance gene are located on pSUP 201-3.

### Cultivation

The cultivation started under aerobic conditions with a pre-culture in a shaker at 37°C and 300 rpm in LB medium (Invitrogen, Darmstadt, Germany). Then, the cells were transferred to a synthetic minimal medium (prepared according to Lara et al., 2008). The medium consists of: 5 g·L^−1^ Glucose, 17 g·L^−1^ K_2_HPO_4_, 5.3 g·L^−1^·KH_2_PO_4_, 2.5 g·L^−1^ (NH_4_)_2_SO_4_, 1 g·L^−1^ NH_4_Cl, 1 g·L^−1^ NaCl, 0.01 g·L^−1^ Thiamine hydrochloride, 1 g·L^−1^·MgSO_4_·7H_2_O, and 1 mL L^−1^ trace elements solution. The trace elements solution contains: 17 g·L^−1^·Zn(CH_3_COO)_2_·2H_2_O, 7 g·L^−1^ Na·EDTA, 1.25 g·L^−1^ CoCl_2_·6H_2_O, 7.5 g·L^−1^ MnCl_2_·4H_2_O, 0.75 g·L^−1^ CuCl_2_·2H_2_O, 1.5 g·L^−1^ H_3_BO_3_, 1.05 g·L^−1^ Na_2_MoO_4_·2H_2_O, 50 g·L^−1^ Fe(III) citrate. All chemicals were purchased from Sigma-Aldrich (Seelze, Germany), Roth (Karlsruhe, Germany), VWR (Darmstadt, Germany), Merck (Darmstadt, Germany), Fluka (Seelze, Germany), and Serva (Heidelberg, Germany). No ampicillin was added in order to avoid antibiotic stress influence on the plasmid production. During the cultivation, samples were successively taken, the cells were harvested by centrifugation (1 mL culture at 10,000 × g), then the supernatant was removed and the pellet was frozen in liquid nitrogen for 10 min and afterwards stored at −80°C. This was done for three different time points during the exponential phase of the cultivation, since we are interested in replication during cultivation. Three biological replicates were taken at each time point. Samples taken during the exponential growth phase are assumed to represent a quasi steady state of the metabolism and plasmid replication and the most representative phase of a pDNA batch production process.

### Growth rate determination

**Figure 1:**
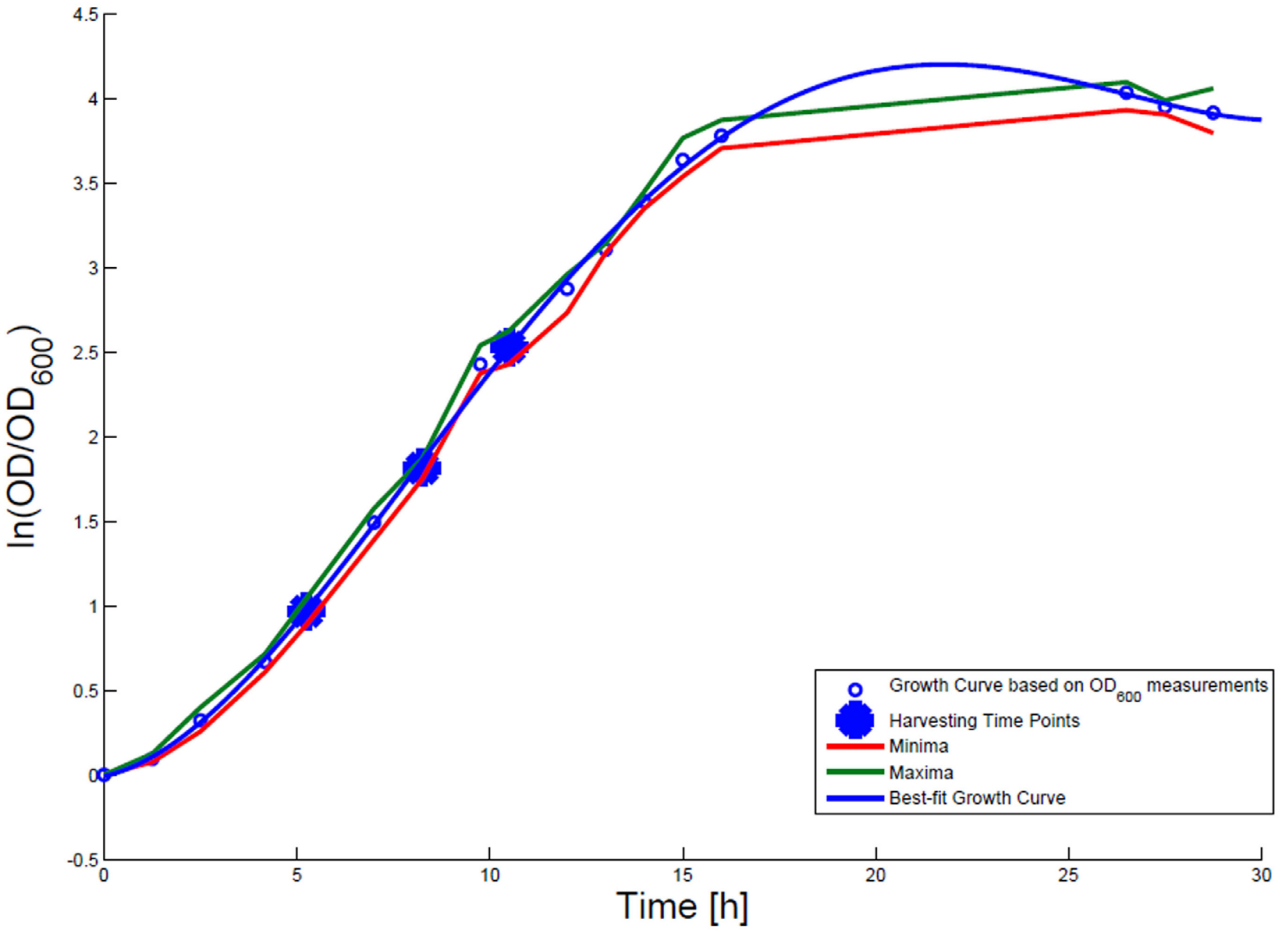
**Growth curve for *E. coli* DH5α-pSUP 201-3**. The growth curve based on OD_600_ measurements are marked by black circles and the appropriate best-fit curve is indicated in blue. The harvesting time points *T*_1_, *T*_2_, and *T*_3_ are presented as bold big points. The minimum–maximum area, within which the measurement values have to reside, is bordered by the red curve (minimal measured OD_600_ values) and the green curve (maximal measured OD_600_ values).

**Figure 2:**
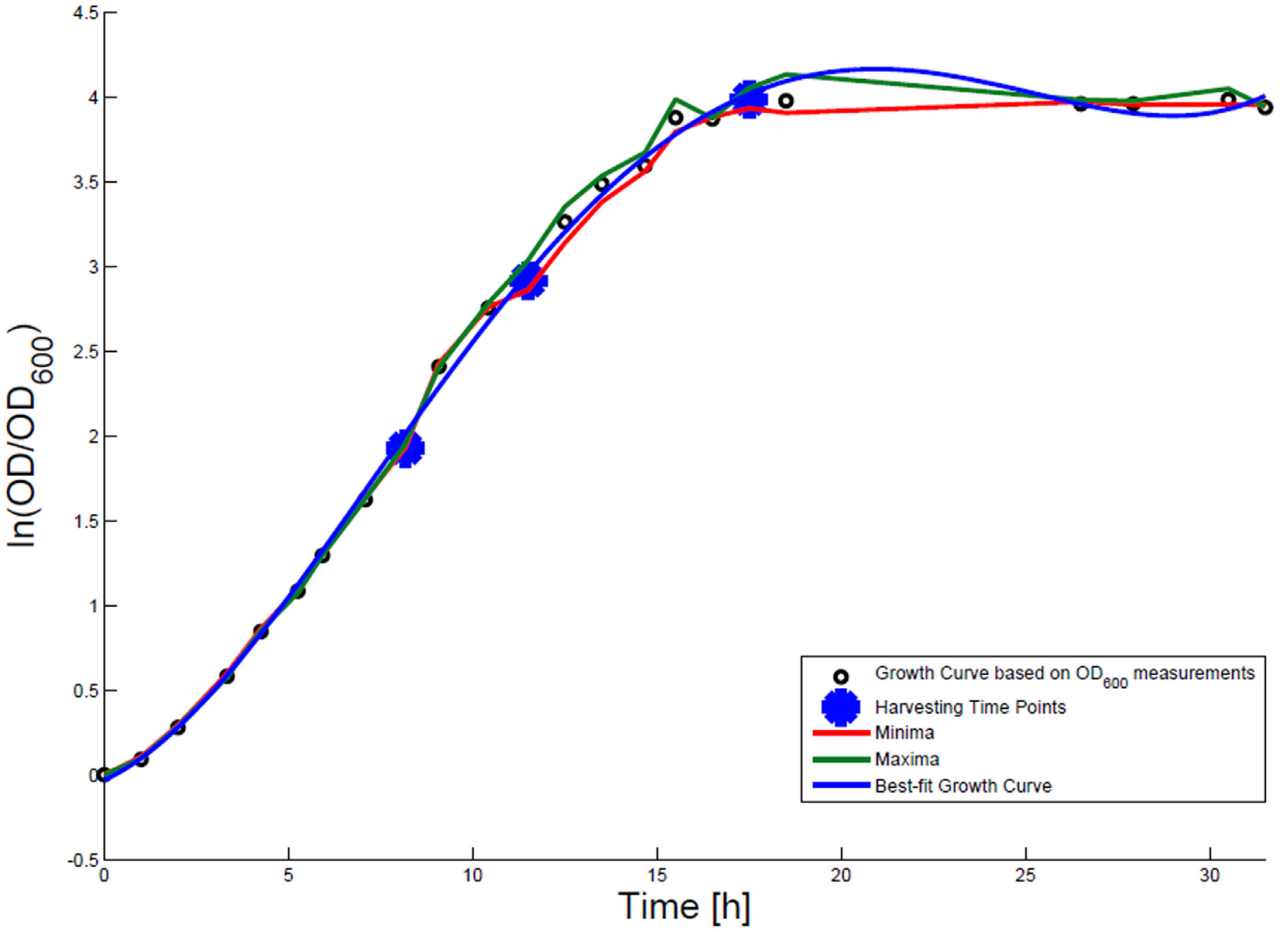
**Growth curve for *E. coli* DH5α-pCMV-lacZ**. The growth curve based on OD_600_ measurements is marked by small black circles and the appropriate best-fit curve is indicated in blue. The harvesting time points *T*_1_, *T*_2_, and *T*_3_ are presented as bold big points. The minimum–maximum area, within which the measurement values have to reside, is bordered by the red curve (minimal measured OD_600_ value) and the green curve (maximal measured OD_600_ value).

For growth rate determination at all measured time points, the cells were cultured in minimal medium and the optical density at 600 nm was measured. Growth curves were generated, where the optical density data as the natural logarithm of the measured OD_600_ values are given on the *y*-axis, normalized to the initial OD_600_ value, and the time is given on the *x*-axis. These growth curves were fitted applying the Matlab function “polyfit.” This function *p* finds the coefficients of a polynomial *p*(*x*) of degree *n* that fits the optical density data stored in a vector *y* best in a least-squares sense, where *p* is a row vector of length *n* + 1 containing the polynomial coefficients in descending powers, *p*(1)*x^*n* + *p*(2)*x^(*n* – 1) + … + *p*(*n*)*x + *p*(*n* + 1). Afterwards a χ^2^-test was applied to test the quality of the fit. The successfully fitted growth curves are shown in Figures 1 and 2.

In the next step, the first derivative *p*′(*x*), which enables the calculation of the growth rate for every time point, was generated.

### Determination of the RNAI and RNAII concentrations

To determine the concentrations of RNAI and RNAII, an internal standard for each of them was synthesized. Therefore, a primer with a T7 RNA polymerase promoter region extension, one for the *rna*I gene and one for the *rna*II gene (Table 1), was designed and a PCR amplification was used to amplify double strand RNAI- and RNAII-DNA. The PCR mixture with a final volume of 25 μL contained 2.5 μL GoTaq reaction buffer, 1.5 μL deoxynucleotides (0.2 mM final concentration), 0.5 μL forward primer and 0.5 μL reverse primer (each with a final concentration of 10 pmol μL^−1^), 0.75 μL MgCl_2_ (final concentration of 1.5 mM), 1 μL GoTaq DNA Polymerase, 0.5 μL pDNA, and nuclease-free water to fill up to 25 μL. All chemicals were obtained from Promega (Mannheim, Germany) except the PCR primers, which were purchased from Metabion (Martinsried, Germany). The PCR was performed in a MJ Research PTC-100 Programmable Thermal Controller from Lab Recyclers (Gaithersburg, USA) applying the following program: an activation step for the hot start Taq polymerase was carried out at 94°C for 2 min, followed by an initial denaturation step at 94°C for 30 s, an annealing step at 64°C for RNAI (67°C for RNAII) and an extension step at 72°C for 20 s. This cycle was repeated 30 times and ended with a final extension step at 72°C for 2 min. The PCR product was purified using the NucleoSpin Extract II Kit from Macherey-Nagel (Düren, Germany) and the concentration was measured spectrophotometrically (NanoDrop2000c spectrometer, Thermo Scientific, Braunschweig, Germany). The PCR product was used as a template for the T7 RNA polymerase (Roche, Mannheim, Germany) according to the manufacturer’s instructions to synthesize RNA. The reaction mixture with a final volume of 20 μL contained 1 mM of each nucleotide (NTP), 2 μL supplied buffer (10×), 40 U T7 RNA polymerase, 20 U Ribolock RNase inhibitor, 1 μg template DNA, and DEPC-treated water to fill up to 20 μL. The mixture was incubated for 2 h at 37°C. The residual template DNA was digested by adding 2 μL DNaseI and incubated 15 min at 37°C. All chemicals were obtained from Fermentas (Schwerte, Germany). The inactivation was done by lithium chloride (LiCl)-precipitation using the following protocol: 2.5 μL LiCl (4M) were added to the 20 μL reaction mixture; in the next step, 75 μL ethanol (>99%) were added. Then, the RNA was precipitated for 30 min at −80°C. After that, for pelleting the RNA, the samples were centrifuged for 15 min at 4°C with 13,000 rpm. The supernatant was discarded and the pellet was washed with 200 μL ethanol (70%). After that it was centrifuged for 15 min at 4°C with 13,000 rpm and the supernatant was discarded. The washing procedure was repeated and after discarding the supernatant, the pellet was dried until the residual ethanol was evaporated. In the last step, the pellet was dissolved in 20 μL nuclease-free water. For generating a calibration for RNAI, a 10-fold serial dilution series ranging from 2 × 10^−1^ to 2 × 10^−6^ ng μL^−1^ was made and analyzed via qRT-PCR. A calibration curve was generated by plotting the cycle threshold values (CT-values) against the time. For the RNAII standard curve, the same procedure was applied. Only the range for the measured 10-fold serial dilution series was different: 2 × 10^−1^to 2 × 10^−7^ ng μL^−1^.

**Table 1:** **Primer sequences to generate internal RNAI and RNAII standards**.

Target	Orientation	Primer (5′–3′)	Length [nt]
*rnaI*	Forward	GAAATTAATACGACTCACTATAGGGACAGTATTTGGTATCTGCGCTC	47
	Reverse	AACCACCGCTACCAGCGG	18
*rnaII*	Forward	GAAATTAATACGACTCACTATAGGGGCAAACAAAAAAACCACCGCTACCA	50
	Reverse	TTTCCATAGGCTCCGCCCCC	20

For sampling the RNA I and RNA II content, cells were cultivated as described above and harvested at each time point. Before harvesting, a dilution series was generated. For different dilutions, 50 μL per sample were plated to determine the cell titer. After doing this, 1 mL of the cell culture was harvested and centrifuged. Then, the supernatant was removed and the pellet was frozen in liquid nitrogen for 10 min and then stored at −80°C. Lysing Matrix B tubes (MP Biomedicals, Heidelberg, Germany) were prepared with 700 μL RLT buffer (RNeasy Plus Mini Kit, Qiagen, Hilden, Germany) with 7 μL β-mercaptoethanol. The filled tubes were incubated on ice for 5 min. The pellet was re-suspended in 200 μL 10 mM Tris-HCl, pH 8, and added to the prepared ice-cold tubes. The cells were lysed using the Hybaid ribolyser for 30 s, level 6.5 m s^−1^, and then incubated on ice for 3 min. After centrifugation at 13,000 rpm and 4°C for 3 min, the supernatant was transferred into a new RNase free reaction tube. The centrifugation step was repeated and again the supernatant was transferred onto the gDNA Eliminator spin column and centrifuged twice at 10,000 rpm, 4°C, and 30 s. The flow through was transferred into a new RNase free reaction tube. The next steps of RNA isolation were performed using the RNeasy Plus Mini Kit (Qiagen, Hilden Germany) according to the protocol Appendix D, starting at step D3 to step D8, with the modification that after step D6 an additional washing step with 500 μL ethanol (80%) was performed. The isolated RNA was measured via quantitative reverse transcription real-time PCR (qRT-PCR) using the Sensi-Mix SYBR No-Rox One Step Kit (Bioline, Luckenwalde, Germany). The reaction mixture was prepared according to the proposed protocol with the modification that the reverse transcription was done with only one primer (RNAI-reverse and RNAII-reverse). The second primer was added just before the beginning of the PCR. As the RNAI molecule is an antisense RNA to the RNAII molecule, we had to stick to our modified protocol in order to avoid that the respective forward primer would bind to both RNA molecules. In this case, both RNA molecules would be reversely transcribed and we would not be able to determine the absolute amount of the particular molecule by PCR. Table 2 shows the used primer sets. The reverse transcription reaction was done in the PCR thermo cycler machine (MJ Research PTC-100 Programmable Thermal Controller from Lab Recyclers, Gaithersburg, MD, USA) at 42°C for 10 min. All real-time-PCR experiments were performed using the Opticon machine (BioRad, München, Germany) according to the following protocol: 10 min at 95°C initial polymerase activation step, 40 cycles [15 s at 95°C, annealing 15 s at 61°C (for RNAI), respectively, 56°C (for RNAII), 15 s at 72°C elongation]. A reaction mixture with a final volume of 20 μL containing 6.2 μL DEPC-treated H_2_O, 10.8 μL Sensi-Mix, 1 μL Primer (RNAI-reverse primer respectively RNAII-reverse primer), and 1 μL RNA was used. After having run the reverse transcription, 1 μL of the second primer was added and the qRT-PCR was started. After each PCR-cycle, the fluorescence was measured for all PCR products. The raw data analysis was carried out with the corresponding Opticon monitor software. Different dilutions of the internal RNA standards were determined via qRT-PCR and the CT-values were calculated. Then the CT-values were plotted against the number of RNA molecules and straight calibration lines for RNAI and RNAII was generated. CT-values were determined as mentioned above for DH5α-low copy RNA samples and DH5α-high copy RNA samples and the appropriate absolute RNA concentration was calculated with the help of the linear regression curve. Then, the RNA amount per cell was calculated for each sample in mol per liter. These values were subsequently used for the theoretical model.

**Table 2:** **Primer sequences to determine the RNAI and RNAII amount via qRT-PCR**.

Target	Orientation	Primer (5′–3′)	Length [nt]
*rnaI*	Forward	TTGGTATCTGCGCTCTGC	18
	Reverse	CAGCGGTGGTTTGTTTGC	18
*rnaII*	Forward	TAACTGGCTTCAGCAGAGCGCAGAT	25
	Reverse	CCTGCCGCTTACCGGATACCTGT	23

### Determination of the plasmid DNA concentrations

The determination of the pDNA concentrations was done by the company Plasmid Factory GmbH & Co. KG (Bielefeld, Germany). The plasmids were isolated by the Plasmid Factory GmbH & Co. KG (Bielefeld, Germany) with a NucleoBond PC20 kit from Macherey-Nagel (Düren, Germany) and the amount of purified pDNA was measured photometrically at the wavelength of 260 nm. Subsequently, the isolated pDNA was separated by agarose gel electrophoresis to check for possible contamination by chromosomal DNA. In case of contaminations, the ratio of chromosomal DNA to pDNA was determined densitometrically using the software LabImage 1D L 340 (Intas Science Imaging GmbH). With this ratio, the amount of pure pDNA in the entire isolated pDNA sample could be determined and with the previously determined cell titers the absolute plasmid concentration in PCN for each harvesting time point was calculated. All measurements were carried out with three biological replicates.

### The theoretical model

**Figure 3:**
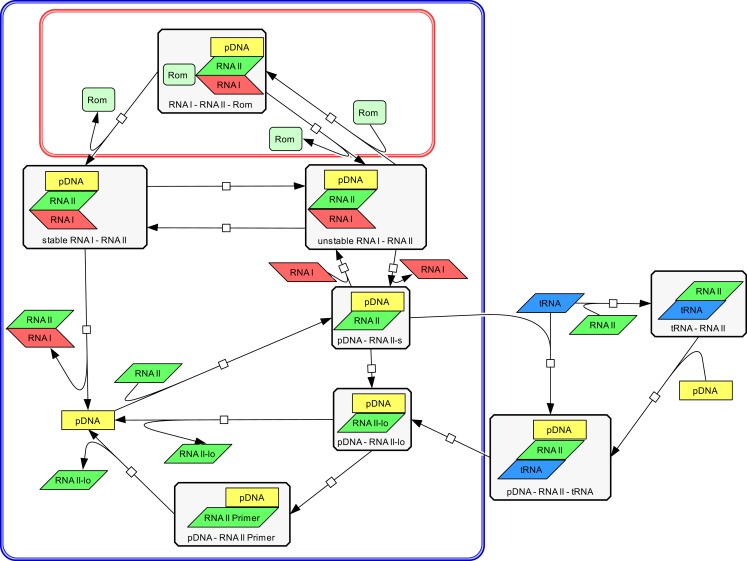
**Structural model of ColE1-like plasmid replication control for low and high copy plasmids as outlined by the CellDesigner software**. The structural model represents an extension of the model proposed by Brendel and Perelson (1993) for ColE1-like plasmid replication control (the corresponding reactions are surrounded by the blue box). The difference between the description of the replication control for a low copy plasmid and for a high copy plasmid is three additional reactions (shown in the red box). These reactions describe the control by the Rom protein and are not active in a high copy plasmid. The reactions outside of the blue box were added within this work and describe the replication control by uncharged tRNA molecules.

The modeling work started with building a structural model to visualize all relevant components and their interactions involved in ColE1-like plasmid replication control. This was done using the CellDesigner software (Ver. 4.1) (Funahashi et al., 2008), in which the replication control of low copy and high copy plasmids are described simultaneously (Figure 3). Note that during low copy plasmid control, three additional reactions representing the Rom protein control are involved. These reactions [see rate equations *v*_1_, *v*_2_, and *v*_13_ in the ordinary differential equation (ODE) system] are marked with a red box in Figure 3 and describe the regulation by the Rom protein, which does not take place in strains carrying the high copy plasmid, due to deletion of the *rom* gene (Yanisch-Perron et al., 1985; Lin-Chao et al., 1992). As mentioned before, this model is an extension of the model presented by Brendel and Perelson (1993), the reactions which originated from their model are marked with a blue box in Figure 3. The remaining reactions describe the regulation by uncharged tRNA molecules under starvation conditions.

### The dynamic model

Based on this structural model, a dynamic model was programed in MATLAB. In that model, each interaction is characterized by a mathematical equation, thereby depicting a kinetic law to define the reaction rates. To describe the dynamics of the substrate concentrations, a system of ODE-System was built, which can be solved numerically, once the initial conditions are set. For replication control by RNAI and RNAII molecules with and without Rom protein, the rate equations recommended by Brendel and Perelson (1993) were used. As for the regulation by uncharged tRNA molecules, we propose kinetic equations based on mass action law. The corresponding parameters are assumed to be stable over time. Clearly, this assumption would not hold, if all growth phases were to be considered. Changing enzyme concentrations along with varying kinetic behavior would long for different models. As our area of interest is the exponential growth phase, we restrict our model to that time interval, within which the parameters stay constant. *In vivo* measurements of plasmid concentrations together with the appropriate growth rates and the RNAI and RNAII concentrations at different time points were incorporated in the dynamic model as initial conditions. The complete ODE-system along with the rate equations can be found in detail in the Supplementary Material.

## Results

### Cultivation and determination of the growth rate for *E. coli* DH5α carrying a low or a high copy plasmid

*Escherichia coli* DH5α-pSUP 201-3 was cultivated in minimal medium and the optical densities of the culture were determined. These measurements were fitted with the Matlab function “polyfit,” which calculates the coefficient parameter set of polynomial coefficients with the lowest residual sum of squares. That is way, a polynomial equation with an order of four was obtained, which describes the bacterial growth for the strain carrying a low copy plasmid. Figure 1 presents the appropriate polynomial growth curve fit for *E. coli* DH5α-pSUP 201-3 together with the measured optical density values and the harvesting time points at which the RNAI, RNAII, and plasmid concentrations were measured. Additionally, the range of the highest and lowest OD_600_ values which were measured for each measuring time point is shown (Figure 1). In analogy to the low copy plasmid strain, the measured optical densities of the high copy strain were fitted with the MATLAB function “polyfit.” The resulting polynomial equation with an order of four describes the bacterial growth of *E. coli* DH5α-pCMV-lacZ (Figure 2). Comparing the measured optical density values with the best-fit curves (Figures 1 and 2), a very good description of the growth behavior for the low copy strain as well as for the high copy strain was obtained. To support this visual impression, χ^2^-tests were applied, which returned reduced χ^2^-values indicating the high fit quality. For both strains, the best-fit curves are located very closely to the measured OD_600_ values and fit well into the area bounded by the highest and lowest OD_600_ values measured between or at least very close to the minimal and maximal determined optical density curve throughout the whole exponential phase (Figures 1 and 2). During this period of cultivation, the theoretical model longs for the respective growth rates: the first derivative of the appropriate polynomial p gives the growth rate μ, for the respective harvesting time point. The calculated growth rates and the appropriate generation times are listed in Table 3 for *E. coli* DH5α-pSUP 201-3 and in Table 4 for DH5α-pCMV-lacZ.

**Table 3:** **Calculated growth rates and generation times for *DH*5α-pSUP 201-3**.

Time point	Harvesting time point [h]	Growth rate [h^−1^]	Generation time [min]
*T*_1_	5.25	0.278	150
*T*_2_	8.25	0.302	138
*T*_3_	10.5	0.283	147

*These parameters are used in the described *in silico* analysis with the dynamic model, when the simulations begin at *T*_1_, *T*_2_, or *T*_3_. In these simulations, the simulation time corresponds to the generation time and the respective growth rate is used as a parameter in the rate equations*.

**Table 4:** **Calculated growth rates and generation times for *DH*5α-pCMV-lacZ**.

Time point	Harvesting time point [h]	Growth rate [h^−1^]	Generation time [min]
*T*_1_	8.17	0.305	136
*T*_2_	11.50	0.256	162
*T*_3_	17.50	0.090	347

*These parameters are used in the described *in silico* analysis with the dynamic model, when the simulations begin at *T*_1_, *T*_2_, or *T*_3_. In these simulations, the simulation time corresponds to the generation time and the respective growth rate is used as a parameter in the rate equations*.

### Determination of the RNAI/RNAII and the pDNA concentrations

The RNAI and RNAII concentrations were determined for three different time points by qRT-PCR and the pDNA concentration was measured photometrically via NanoDrop and checked by agarose gel electrophoresis. This was carried out for the low copy strain, *E. coli* DH5α-pSUP 201-3, and for the high copy strain, *E. coli* DH5α-pCMV-lacZ, with three biological replicates. After qRT-PCR, the RNA concentrations were calculated by generating straight calibration lines from the RNAI and RNAII standards through linear regression. Regarding the results of *E. coli* DH5α-pSUP 201-3, a decrease of the RNAI and RNAII concentrations with proceeding growth of the bacterial culture was observed (Table 5). Additionally, it was apparent that at every harvesting time point, there are always more free RNAI molecules than RNAII molecules in the cell. In contrast to the RNA concentrations, the measured plasmid concentration per cell was stable over all three harvesting time points. PCNs of 46–48 were calculated for each harvesting time point (Table 5). Regarding the determined PCNs of *E. coli* DH5α-pCMV-lacZ, an increase in plasmid concentrations with proceeding growth of the bacterial culture was observed (Table 6). For harvesting time point *T*_1_, a PCN of 1514 per cell was determined, while at *T*_2_ a PCN of 2403 was found. At *T*_3_, 5805 plasmids per cell were measured. Cooper and Cass (2004) reported a PCN of 500–700 for pUC derivatives, so compared to the literature, the measured PCNs are very high. For this reason, we double checked the high magnitude of some of these values by qRT-PCR. The PCNs obtained by this analysis were in the same order of magnitude as the previously determined values. Considering the RNA concentrations per cell, fluctuations in the RNAI concentrations were observed in contrast to measurements for *E. coli* DH5α-pSUP 201-3, while the RNAII concentrations increased with proceeding growth of the bacterial culture (Table 6). The highest RNAI concentration was measured at *T*_2_ with 1086 ± 298 RNAI molecules per cell and the highest RNAII concentration was found at *T*_3_ with 75 ± 10 molecules per cell. If these RNA concentrations are compared to the RNA concentrations measured for *E. coli* DH5α-pSUP 201-3, it is apparent that at every harvesting time point, the amount of free RNAI and RNAII molecules in *E. coli* DH5α-pCMV-lacZ is higher than those in *E. coli* DH5α-pSUP 201-3.

**Table 5:** **Measured RNAI-, RNAII-, and plasmid concentrations for DH5α-pSUP at the three harvesting time points, depicted in Figure 1**.

Time point	RNAI [molecules/cell]	RNAII [molecules/cell]	PCN [molecules/cell]
*T*_1_	34 (±7)	3 (±0.9)	46 (±26)
*T*_2_	17 (±5)	1 (±0.4)	48 (±30)
*T*_3_	6 (±0.7)	0.44 (±0.1)	46 (±17)

**Table 6:** **Measured RNAI-, RNAII-, and plasmid concentrations for DH5α-pCMV-lacZ at the three harvesting time points, depicted in Figure 2**.

Time point	RNAI [molecules/cell]	RNAII [molecules/cell]	PCN [molecules/cell]
*T*_1_	541 (±51)	22 (±2)	1514 (±1301)
*T*_2_	1086 (±298)	64 (±4)	2403 (±713)
*T*_3_	345 (±203)	75 (±10)	5806 (±4828)

For determination of the number of RNAI/RNAII molecules per cell as well as the PCN, the mean *M* (*i* = 1, 2, 3) for each biological replicate was calculated applying

$$M_{i}=\frac{1}{N_{i}}\times \sum_{j=1}^{N_{i}}m_{\mathrm{ji}}\left(i=1,\;2,\;3\right).$$

The respective SD was determined by

$${\mathrm{SD}}_{i}=\sqrt{\frac{1}{N_{i}}\times {\sum_{j=1}^{N_{j}}\left(m_{\mathrm{ji}}=M_{i}\right)}^{2}}.$$

### The structural model of ColE1-like plasmid replication control

Using the CellDesigner software (Funahashi et al., 2008), a structural model for the ColE1-like plasmid replication control was built (Figure 3). The involved elements and their interactions are given to describe the ColE1-like plasmid replication control in high- and low copy plasmids simultaneously. Three additional reactions make the difference between low copy plasmid replication control and high copy plasmid replication control. These reactions represent the control by the Rom protein and are marked with a red box in Figure 3. In case of a high copy plasmid replication, the control level by the Rom protein does not take place due to the deletion of the *rom* gene (Yanisch-Perron et al., 1985; Lin-Chao et al., 1992). Since the model proposed in this study is an extension of the model presented by Brendel and Perelson (1993), the reactions which came from their model are marked by a blue box in Figure 3. The remaining reactions that were added in this study describe the regulation by uncharged tRNA molecules under starvation conditions. Regarding the replication control by uncharged tRNA molecules, there are two possibilities for the uncharged tRNA’s (tRNA) to act: the uncharged tRNA molecules can bind to plasmid bound RNAII molecules or they can bind to free RNAII molecules and afterwards build a complex with the pDNA. In both cases, the binding of the inhibitory RNAI molecules is prohibited and the elongation of the RNAII-primer can occur. According to Brendel and Perelson (1993) the synthesis and degradation reactions of RNAI, RNAII, and the Rom protein were included. Since the model proposed in this study contains the regulatory level of uncharged tRNA molecules, it was necessary to add also the synthesis and degradation reactions of uncharged tRNA. These synthesis and degradation reactions are not shown in the structural model depicted in Figure 3, they are found in the Supplementary Material.

The plasmid replication depicted in Figure 3 starts with the transcription of a preprimer RNAII molecule from a region 555 bp upstream of the origin of replication. This RNAII transcript has a length of 100–360 nt and persistently hybridizes with a region close to the origin of replication (pDNA–RNAII-s) (Tomizawa, 1986). At that step, there are three possible scenarios for the proceeding of the replication process: the first one is that a regulatory RNA molecule, named RNAI, binds to the hybridized RNAII molecule. The regulatory RNAI molecule is encoded on the opposite strand of that one on which RNAII is encoded. The synthesis of RNAI begins 445 nt upstream of the origin of replication and proceeds in the antisense direction compared to the RNAII synthesis. Since the antisense RNAI transcript is complementary to RNAII, it is able to bind the plasmid-RNAII hybrid and forms a so-called kissing complex (pDNA–RNAII–RNAI unstable). When this transient complex is built, the elongation of RNAII is blocked (Tomizawa, 1986). The second scenario is that the hybridized RNAII molecule is elongated to a length >360 bp before an RNAI molecule could bind (pDNA-RNAII-lo). In that case, RNAII is cleaved by an enzyme named RNase H. This enzyme releases the 3’OH group, so the modified RNAII molecule can act as a primer (pDNA–RNAII-Primer). In the following reaction, the RNAII-Primer will be elongated by the DNA polymerase I and the pDNA will be doubled (Itoh and Tomizawa, 1980). As in the model of Brendel and Perelson (1993), we assume that the concentration and the activity of RNase H remain constant. Additionally, it was assumed that RNAI, RNAII, the Rom protein, and uncharged tRNA molecules move freely through the cytoplasm.

The third scenario is the binding of a free uncharged tRNA to the plasmid bound RNAII (pDNA–RNAII–tRNA). When an uncharged tRNA is bound to RNAII, the RNAI molecules cannot bind anymore, but RNAII can still be cleaved and elongated and serves in this way as a primer (pDNA–RNAII-Primer) (Grabherr and Bayer, 2002). It is proposed in this model that not only plasmid bound RNAII can interact with an uncharged tRNA molecule but also free RNAII molecules can be bound by an uncharged tRNA (RNAII–tRNA). In that case, the RNAII–tRNA complex binds to the template DNA and a complex composed of a plasmid, RNAII, and an uncharged tRNA molecule is built (pDNA–RNAII–tRNA). The RNAII molecule will be elongated and becomes a primer, so the plasmid replication starts. This third scenario was not considered in the model of Brendel and Perelson. Considering the case that the primer could not be formed because of binding of the RNAI molecule to the plasmid bound RNAII, an unstable complex between the plasmid, RNAII and RNAI is built (pDNA–RNAII–RNAI unstable). This complex could become stable, modeled by a rate equation with the rate constant *k*_10_ (Brendel and Perelson, 1993). Having a low copy plasmid, this unstable complex could be stabilized by the Rom protein. As proposed in the model from Brendel and Perelson (1993), we assume that therefore the Rom protein binds to the unstable complex and builds a transient complex (pDNA–RNAII–RNAI–Rom). This transient complex then is converted into a stable complex between of plasmid, RNAII, and RNAI (pDNA–RNAII–RNAI stable). Since the Rom protein is not present in a high copy plasmid, the reactions with Rom participation are missing (Figure 3). In addition to the reactions presented in the structural model in Figure 3, we consider the synthesis and degradation reactions of RNAI, RNAII, uncharged tRNA, and of the Rom protein (Rom).

### The dynamic model of ColE1-like plasmid replication control

In the dynamic model, the *in vivo* reaction kinetics of each interaction presented in the structural model (Figure 3) are described by applying mass action law kinetics. In addition to those reactions shown in Figure 3, the reaction rates of the synthesis and degradation reactions of RNAI, RNAII, uncharged tRNA, and Rom protein were specified by mathematical equations. Due to the assumption that the kinetic parameters do not change through the exponential growth phase, they remain constant for every growth rate. The dynamics of the substrate concentrations are described by ODEs. In general, such an ODE reads as follows:

(1) $$\frac{d\left[S\right]}{\mathrm{dt}}=v_{1}+\ldots +v_{n}-\mu \times \left[S\right]$$

with [*S*] representing the substrate concentration, *v*_i_ (*i* = 1 … *n*) describing the involved reaction rates, *t* the time, and μ the specific growth rate. In every ODE a term, where the negative growth rate is multiplied with the substrate concentration (−μ … [*S*]), was included. The application of the −μ … [*S*] term was proposed by Brendel and Perelson (1993) to account for the dilution effect due to cell growth. The dilution effect describes that the distribution of intracellular compounds to the daughter cell after cell division depends on the growth rate. In case of a fast growing cell, the intracellular volume will increase faster than the concentration of intracellular compounds. As a consequence, the daughter cell shows a lower concentration of intracellular compounds than the mother cell. A lower dilution effect was observed in slow growing cells. The reason for that is that a slow growing mother cell has more time to synthesize her intracellular compounds before it will divide into two daughter cells. Thus, the characteristics of the dilution effect depend on the growth rate. The ODE system is applied to simulate the regulation of ColE1-like plasmid replication. It was set up based on the structural model by applying mass action law together with the dilution term. The model implies 15 metabolites (*s*_i_ = 1, … , 15) whose interactions are described by 26 rate equations (*v*_i_ = 1, … , 26) containing 26 kinetic constants *k*_i_. This means that all reactions visualized in the structural model by a reaction arrow are described mathematically by a rate equation (*v*_1_, … , *v*_17_). In addition to the visualized reactions in the structural model, synthesis and degradation reactions of Rom protein, RNAI/RNAII, and uncharged tRNA together with the reaction for complex formation of RNAI and RNAII are described by rate equations (*v*_18_, … , *v*_26_). The model proposed in this work is a minimal model which includes the plasmid replication control by two regulatory RNA molecules (RNAI and RNAII), the control by Rom protein as well as the replication control by uncharged tRNA molecules. It comprises no reaction not involved in these levels of replication control. The mentioned 26 rate equations are necessary and sufficient to simulate the ColE1-like plasmid replication control, because they describe all relevant elements to investigate the proposed hypothesis.

The whole ODE-system and the 26 rate equations can be found in the supplement. The parameter values *k*_i_ (*i* = 1, … , 26) used in the rate equations with the respective source are listed in Table 7. After defining all kinetic constants, the ODE-system could be solved numerically by applying a MATLAB solver. As initial conditions, the experimentally determined RNAI and RNAII concentrations together with 50% of the respective determined appropriate plasmid concentrations and the appropriate measured growth rates were used. The motivation for taking the half of the measured plasmid concentration value is the assumption that the measured plasmid concentration is equal to the plasmid concentration directly before cell division and that the pDNA will be doubled during one generation time. All unknown substrate concentrations were assigned the zero value, because it is assumed that the internal pool of these substances has to be synthesized and is comparatively low at the beginning of the investigation. Once the above-mentioned parameters and initial concentrations were included into the dynamic model, it was used for *in silico* analysis of the ColE1-like plasmid replication control.

**Table 7:** **Parameter for *in silico* analysis of ColE1-like plasmid replication control**.

Reaction kinetic	Constant	Low copy plasmid	High copy plasmid	Source
1	*k*_1_ [M^−1^·min^−1^]	1.7·10^8^	Not available	^a^^,^^-^
2	*k*_2_ [min^−1^]	0.17	Not available	^a^^,^^-^
3	*k*_3_ [M^−1^·min^−1^]	1.02·10^8^	3.05·10^6^	^c^^,^^c^
4	*k*_4_ [min^−1^]	48	20	^a^^,^^c^
5	*k*_5_ [min^−1^]	12	12	^a^^,^^a^
6	*k*_6_ [min^−1^]	4.3	4.3	^a^^,^^a^
7	*k*_7_ [min^−1^]	3.8	4.19	^f^^,^^f^
8	*k*_8_ [min^−1^]	4.3	4.3	^a^^,^^a^
9	*k*_9_ [M^−1^· min^−1^]	0.25	0.25	^a^^,^^a^
10	*k*_10_ [min^−1^]	44	44	^a^^,^^a^
11	*k*_11_ [min^−1^]	0.085	0.085	^a^^,^^a^
12	*k*_12_ [min^−1^]	17	17	^a^^,^^a^
13	*k*_13_ [min^−1^]	34	Not available	^a^^,^^-^
14	*k*_14_ [min^−1^]	1.8·10^5^	1.8·10^5^	^b^^,^^b^
15	*k*_15_ [M^−1^·min^−1^]	1.8·10^5^	1.8·10^5^	^b^^,^^b^
16	*k*_16_ [min^−1^]	12	12	^g^^,^^g^
17	*k*_17_ [min^−1^]	0.25	0.25	^g^^,^^g^
18	*k*_18_ [min^−1^]	6	6	^a^^,^^a^^,^^d^
19	*k*_19_ [min^−1^]	0.25	0.25	^a^^,^^a^^,^^d^
20	*k*_20_ [min^−1^]	4	Not available	^a^^,^^-^
21	*k*_21_ [min^−1^]	0.35	0.35	^a^^,^^a^
22	*k*_22_ [min^−1^]	0.35	0.35	^a^^,^^a^
23	*k*_23_ [min^−1^]	0.14	Not available	^a^^,^^-^
24	*k*_24_ [M·min^−1^]	7.99·10^−6^	7.99·10^−6^	^e^^,^^e^
25	*k*_25_ [M^−1^·min^−1^]	1.02·10^8^	3.05·10^6^	^g^^,^^g^
26	*k*_26_ [min^−1^]	0.35	0.35	^c^^,^^c^

*^-^No literature data is found for this value*.

*^a^Brendel and Perelson (1993)*.

*^b^Wang et al. (2002) with the assumption that RNAII molecules are bound by tRNA molecules with the same kinetic constant like RNAI molecules bind to RNAII molecules in reaction 3*.

*^c^This work (parameter fitting)*.

*^d^k_24_ and k_19_ were calculated with an assumed RNA polymerase-transcription rate of 50 nt s^−1^ (von Hippel et al., 1984)*.

*^e^k_24_ was calculated with an assumed transcription rate of 42 nt s^−1^ [Gotta et al. (1991) and Klumpp (2011)]*.

*^f^k_7_ was calculated with an assumed DNA-polymerase-elongation rate of 42 bp s^−1^ (Alberts et al., 2007) under allowance of plasmid size*.

*^g^This work (estimated)*.

### Simulations of the ColE1-like plasmid replication control – simulations for parameter fitting

Since some kinetic values are unknown so far, they had to be either estimated or determined via parameter fitting. To obtain reasonable fits for the constants *k*_3_, *k*_4_, and *k*_26_ (Table 7), the experimentally determined data of harvesting time point *T*_2_ were used for the low copy plasmid as well as for the high copy plasmid. Obviously, this data set was not considered for later validation. In all simulations, one single cell was considered. The simulation time corresponds to the generation time, which was calculated using the respective growth rates determined for each harvesting time point. The simulations for parameter fitting and validation for DH5α-pSUP 201-3 began at the particular harvesting time point and ran for the period of one cell duplication (i.e., simulation time = generation time), where one single cell was considered. Regarding the results for *T*_3_, for example, the simulation began at time point *T*_3_ and the simulation time was 147 min. In biological sense, this means that at *T*_3_ one single cell starts to grow, divides after 147 min and ends up with a PCN of 49, directly before the next cell division. The results of the parameter fitting and validation for *E. coli* DH5α-pSUP 201-3 are depicted in Table 8 in terms of simulated PCNs and experimentally determined PCNs. Besides the results assigned to time point *T*_2_, which can be regarded as the outcome of the fitting procedure, the simulation results for the remaining time points *T*_1_ and *T*_3_ meet the experimental outcome very well. These results were achieved without any additional fitting steps, just with the parameters determined for time point *T*_2_, which are summarized in Table 7 (fitted parameters are assigned the letter “c”). Comparing the simulated PCNs and the measured PCNs of *E. coli* DH5α-pSUP 201-3 and *E. coli* DH5α-pCMV-lacZ (Table 9), respectively, one can conclude that the dynamic model supplied with this data set can sufficiently explain the bacterial behavior for different cultivation time points. Comparing the simulated PCNs and the measured PCNs of *E. coli* DH5α-pSUP 201-3, it could be concluded that the dynamic model is able to reproduce the experimentally measured PCNs in an appropriate way. Looking at the simulated PCNs for *E. coli* DH5α-pCMV-lacZ, it could be observed that in this case the model is also able to reproduce the experimentally determined PCNs in an appropriate way (Table 9). The simulated PCNs reside within the margin represented by the SD. To sum up, the simulation of the plasmid replication control for all harvesting time points is possible and the model is able to reproduce the PCN in the requested correctness.

**Table 8:** **Results of the *in vitro* and *in silico* determined PCNs for DH5α-pSUP**.

Time point	Measured pDNA [molecules/cell]	Simulated pDNA [molecules/cell]
*T*_1_	46 ± 26	49
*T*_2_	48 ± 30	47
*T*_3_	46 ± 17	49

*The *in vitro* determined PCNs are the results of the plasmid concentration measurements done by the Plasmid Factory (Bielefeld, Germany) and the *in silico* determined PCNs are calculated by the dynamic model. As initial conditions for the simulations, the experimentally determined data for the three harvesting time points *T*_1_, *T*_2_, and *T*_3_ were chosen. To these belong the measured RNAI-, RNAII-, and plasmid concentrations (Table 5) and the respective growth rates (Table 3). The simulation time was equal to the generation time (Table 3)*.

**Table 9:** **Results of the *in vitro* and *in silico* determined PCNs for DH5α-pCMV-lacZ**.

Time point	Measured pDNA [molecules/cell]	Simulated pDNA [molecules/cell]
*T*_1_	1514 ± 1301	1718
*T*_2_	2403 ± 713	2405
*T*_3_	5806 ± 4828	5421

*The *in vitro* determined PCNs are the results of the plasmid concentration measurements done by the Plasmid Factory (Bielefeld, Germany) and the *in silico* determined PCNs are calculated by the dynamic model. As initial conditions for the simulations, the experimentally determined data for the three harvesting time points *T*_1_, *T*_2_, and *T*_3_ were chosen. To these belong the measured RNAI-, RNAII-, and plasmid concentrations (Table 6) and the respective growth rates (Table 4). The simulation time was equal to the generation time (Table 4)*.

### Predictive simulations

In this study, a dynamic model was developed to investigate the effect of uncharged tRNA molecules on the ColE1-like plasmid replication control. From this, the question arises if tRNA molecules can be modified in such a way that they cannot be charged any longer, and could we envisage an increase in plasmid production? In general, a high amount of uncharged tRNA molecules can be observed when the cell is afflicted with amino acid starvation. Under that condition, the protein synthesis is negatively affected and the majority of tRNA molecules in the cytoplasm are uncharged. Wróbel and Węgrzyn (1998) could show that a high amount of uncharged tRNA molecules is connected with an increase in the PCN (Wróbel and Węgrzyn, 1998). Thus, for plasmid production, starvation conditions would be advantageous. There is just one single problem: for a long-term production process, these conditions are not applicable, because the cells will not survive until the plasmid production cultivation is finished. One possible solution could be the overexpression of a tRNA gene. But in that case, there is a certain probability that the higher amount of uncharged tRNA will be rapidly charged by the amino-acyl- tRNA synthetases before they could influence the plasmid replication. To overcome this problem, a modification of the tRNA molecule is required, which would preserve the charging and still positively influence the plasmid replication. In that case, the cell would be still vital under non-starvation conditions. But could such modified tRNA molecules actually push the plasmid replication? Therefore, the dynamic model was established, which enables to investigate how the plasmid replication control would behave, if a gene encoding a modified tRNA molecule was introduced into the genome.

**Figure 4:**
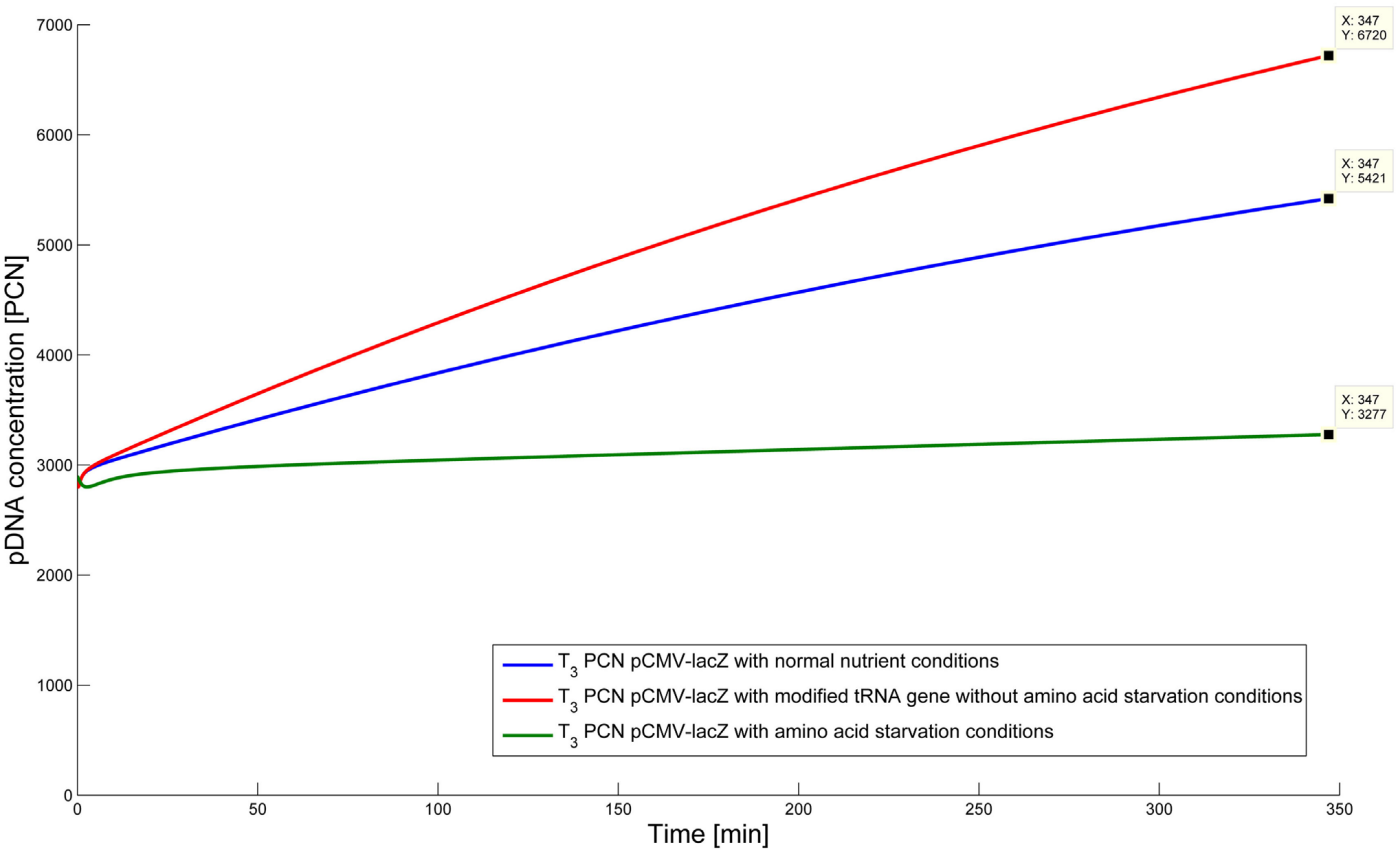
**Simulation of the plasmid replication control for *E*. *coli* DH5α-placZ, beginning at time point *T*_3_, for the following three different growth conditions: blue, growth with normal nutrient conditions; green, growth with amino acid starvation conditions, red, growth with modified tRNA gene without amino acid starvation conditions**.

To test the hypothesis that an uncharged tRNA would increase the PCN, the ColE1-like plasmid replication control of *E. coli* DH5α-pCMV-lacZ was simulated for the following three conditions: growth under normal nutrient conditions, growth under amino acid starvation conditions, and growth under the influence of an inserted gene encoding for a modified tRNA under normal nutrient conditions. *E. coli* DH5α-pCMV-lacZ was chosen, since for plasmid production usually high copy plasmids are used. The simulations could also be conducted for *E. coli* DH5α-pSUP 201-3, leading qualitatively to the same result (data not shown). The simulations were done for all three conditions, beginning at *T*_3_, for a period of one cell duplication [i.e., simulation time = generation time (*T*_3_)]. One single cell is considered, which starts to grow at time point *T*_3_ and divides after 347 min. The initial conditions were the experimentally determined data at *T*_3_ (see Tables 4 and 6), which includes the RNAI- and RNAII concentrations together with half of the respective determined plasmid concentrations and the appropriate measured growth rate. All unknown substrate concentrations were assigned the value 0. This means that the simulations consider one single cell, which starts to grow at time point *T*_3_ and divides after 347 min. In the moment of cell division, the simulation stops, so the daughter cells are not considered anymore. The time point *T*_3_ was chosen exemplarily, because at this time point the highest PCN was measured. Of course, it is possible to do these simulations just as well for *T*_1_ and *T*_2_. The simulation results of the three mentioned conditions are graphically shown in Figure 4. The first simulation (Figure 4) considers growth conditions with normal nutrient supply. To describe this situation mathematically, the kinetic constant of uncharged tRNA synthesis (*k*_24_) was multiplied with the factor 0.01 to keep the amount of free uncharged tRNA molecules very low, because the amino acids supply is sufficient, so the amount of free uncharged tRNA molecules is very low. The factor 0.01 was chosen due to the assumption that under sufficient nutrient supply, only 1% of the tRNA molecules in the cell are uncharged. The *in silico* analysis showed that for a cell, growing under normal nutrient conditions, the PCN undergoes an increase to about 2523 (5421 plasmids at *T*_3_ – initial PCN = 2523). The second simulation (Figure 4) was done for a cell, which grows under amino acid starvation conditions. Here, the assumption was made, that as a consequence of insufficient nutrient supply, the intracellular protein concentrations are decreased and so all reactions run with only 10% reaction velocity (estimated). Mathematically spoken, every kinetic constant and the growth rate were multiplied by the factor 0.1. Furthermore, the factor of 0.01 for *k*_24_ was replaced by 1, because of the high amount of uncharged tRNA molecules under amino acid starvation. The second simulation predicts that within a period of cell duplication, one single cell produces only 379 plasmids. In the third simulation (Figure 4), the cells grow under normal nutrient supply with a high amount of modified tRNA molecules, which were encoded by an inserted modified tRNA gene. As for the mathematical description, this means that all reactions are running at 100%, because there is no amino acid starvation. Furthermore, the factor 0.01 for the kinetic constant *k*_24_ was again replaced by 1, since there are a lot of free modified tRNA molecules in the cell. Under these conditions, the model predicts a plasmid production of 3822 plasmids for one single cell within a period of cell duplication. Comparing all three simulations (Figure 4), it is apparent, that the plasmid production is lowest under insufficient nutrient supply. The highest plasmid production is predicted for the case when a gene, which encodes a modified, uncharged tRNA, is introduced into the genome.

## Discussion

In the last 10 years, the number of clinical trials in the field of human gene therapy has increased from 500 to 1500 (Prazeres, 2011). One can assume that once the clinical trials are completed and plasmid biopharmaceuticals enter the market, the demand of pDNA will increase. Since ColE1-type plasmids are often the basis for DNA vaccines or gene therapy products, it is important to understand how their replication is regulated (Prather et al., 2003). The dynamic model presented here is a valuable contribution to the modeling work done in this field, since it is more comprehensive than previous models (Ataai and Shuler, 1986; Bremer and Lin-Chao, 1986; Keasling and Palsson, 1989a,b; Brendel and Perelson, 1993; Wang et al., 2002). It extends the model proposed by Brendel and Perelson (1993), because it does not only incorporate the regulation through inhibitory RNAI molecules, but it considers also the control by uncharged tRNA molecules. Some previous models have focused on the PCN deviation from steady states derived from RNAI and RNAII concentrations (Paulsson et al., 1998), plasmid stability or probability of plasmid loss (Paulsson and Ehrenberg, 1998), as well as the formation of stable RNAII structures (Gultyaev et al., 1995). However, the mentioned reports did not show the effect of RNAI, RNAII, or uncharged tRNA on PCN linked to cell growth. Thus, the underlying reaction network of the proposed model is more detailed and the number of application conditions is increased. The simulation data are confirmed by *in vitro* measurements, obtained at three different time points. Since the data for each time point were measured for each time point in the same strain, they can be treated as homogeneous data sets. Many published dynamic models are based on heterogeneous data sets that consist of experimental data obtained from different strains and partly from different organisms (Klumpp, 2011). These heterogeneous data sets are not optimal for modeling, because usually the model should help to investigate a specific organism, so data measured in a foreign organism might be unsuitable. In comparison to other growth rate measurements both strains used in this work grow very slowly. This slow growth could be reasoned by the usage of minimal medium for cultivation, because it contains only essential additives. On LB-medium both strains show the typical growth behavior for *E. coli* DH5α.

The intracellular RNAI and RNAII concentrations were calculated for *E. coli* DH5α-pSUP 201-3 and *E. coli* DH5α- pCMV-lacZ via qRT-PCR with the aid of straight calibration lines. The intracellular RNAI- and RNAII concentrations measured for *E. coli* DH5α-pSUP 201-3 decrease with proceeding growth of the bacterial culture. Furthermore, it was observed that there are more free RNAI molecules than RNAII molecules at each harvesting time point. The RNA measurements of this study are similar to the RNAI- and RNAII measurements done by Brenner and Tomizawa (1991) 23 years ago. Brenner and Tomizawa (1991) quantified the RNAI and RNAII concentrations densitometrically for an *E. coli* C600 derivative (SA791) strain carrying a pCer plasmid or a pΔP4 plasmid (both low copy plasmids) applying quantitative probe protection experiments. They measured an average RNAII molecule number of 1.9 ± 0.9 to 3.7 ± 1.0 per cell and an average RNAI molecule number of 333 ± 49 to 499 ± 36 per cell (Brenner and Tomizawa, 1991). The RNAI concentrations determined by Brenner and Tomizawa (1991) are an order of 10 higher than the molecule numbers measured in this work. This could be reasoned by the different measuring methods. Possibly, the qRT-PCR is a more sensitive method compared to the quantification by quantitative probe protection experiments. Furthermore, Brenner and Tomizawa (1991) used a minimal medium supplemented with non-essential additives for cultivation, which could also have influenced the RNAI concentration. In comparison to *E. coli* DH5α-pSUP 201-3, the RNAI- and RNAII concentrations measured for *E. coli* DH5α- pCMV-lacZ do not decrease with proceeding growth of the bacterial culture. On the contrary, the intracellular RNAII concentrations increased with proceeding cultivation. Regarding the RNAI amount, the highest RNAI concentration was measured at time point *T*_2_ and the lowest at time point *T*_3_. Since there are no comparative values published so far, the measured RNA concentrations of *E. coli* DH5α-pCMV-lacZ cannot be compared with previously determined values. For *E. coli* DH5α-pSUP 201-3, PCNs of 46 or 48 were measured at all three time points. The published PCNs for pBR322 plasmids are 15–20 plasmids per cell (Cooper and Cass, 2004), so the measured PCNs for *E. coli* DH5α-pSUP 201-3 are around two times higher. But considering the SDs of the measurements of this study, it could be seen that the PCNs measured by Cooper and Cass (2004) reside within the same range, defined by the SDs. Again the difference could be explained by the cultivation in minimal medium with no non-essential supplements used in this work, because the *E. coli* DH5α-pSUP 201-3-cells grew very slowly and a slow growth promotes the intracellular plasmid concentration (Bremer and Lin-Chao, 1986). Taken as a whole, the plasmid amount per cell of *E. coli* DH5α-pSUP 201-3 is stable within all three time points. In contrast to the low copy plasmid strain *E. coli* DH5α-pSUP 201-3, the plasmid concentration of *E. coli* DH5α-pCMV-lacZ increases with progressing growth of the bacterial culture. The highest plasmid concentration was measured at time point *T*_3_ with 5806 ± 4828 plasmids per cell. It can be assumed that the increasing RNAII concentration is responsible for the increase of the PCN, because more RNAII molecules are available to serve as a primer. Comparing these measured PCNs with literature values shows that they are very high. For pUC plasmids, copy numbers of 500–700 were reported (Cooper and Cass, 2004). To address these differences, plasmid numbers were confirmed by qRT-PCR, resulting in the same order of magnitude. As for *E. coli* DH5α-pSUP 201-3, the minimal medium and the slow growth could be responsible for that high plasmid concentrations.

After the experimental data had been introduced to the dynamic model, the unknown parameters were partly obtained by parameter fitting. After comparing simulation results with our own experimental as well as literature data and achieving a satisfying validation, the model was ready to be used to do *in silico* analysis. The hypothesis that modified tRNA molecules would have a similar pushing effect on plasmid production like uncharged tRNA molecules have under starvation conditions was tested. The simulations showed that under amino acid starvation, the smallest amount of plasmids is produced. As for the effect of modified tRNA molecules on plasmid production, the model predicts an increase compared to the plasmid production under normal nutrient conditions. Thus, the insertion of a gene encoding modified uncharged tRNA molecules would have a positive effect on the plasmid production. Since the hypothesis is confirmed by *in silico* analysis with the established model, the next step will be to concentrate on the design of a modified uncharged tRNA gene. This gene should encode for a modified tRNA molecule, which will not be recognized by an amino-acyl synthetase and therefore will not be charged anymore. Additionally, all elements influencing the ColE1-like plasmid replication control have to maintain functionality.

## Conclusion

In this study, a dynamic model of ColE1-like plasmid replication control for a low copy plasmid and a high copy plasmid was established. The model comprises the replication control by regulatory RNA molecules and by uncharged tRNA molecules. The big advantage of this model is that it is confirmed by *in vitro* measurements, which were done for the same strains and at three different time points. The established model was used to simulate the plasmid replication control for the period of one cell duplication, where one single cell was considered. Nevertheless, the model can also be used to predict the plasmid replication control for more than one period of cell duplication, which could be conducted for constant growth conditions as well as for varying growth conditions. The simulations done with the dynamic model within this work predict that inserting a gene encoding modified uncharged tRNA molecules would increase the PCN. Since the overall aim is to improve the plasmid production to accommodate the prospective demand on pDNA, this predicted effect of modified tRNA molecules is very important. For that reason, further investigation should be concentrated on the construction of such a modified uncharged tRNA gene.

## Conflict of Interest Statement

The authors declare that the research was conducted in the absence of any commercial or financial relationships that could be construed as a potential conflict of interest.

## Supplementary Material

The Supplementary Material for this article can be found online at http://journal.frontiersin.org/article/10.3389/fbioe.2015.00127
