# Supplementary material for: ColE1-Plasmid Production in Escherichia coli: Mathematical Simulation and Experimental Validation
Source: Front Bioeng Biotechnol. 2015 Sep 1;3:127. doi: 10.3389/fbioe.2015.00127 (PMC4555960; doi:10.3389/fbioe.2015.00127)
Supplement: Supplementary file 2 [file Data_Sheet_2.DOCX]

**Supplemental material**

**The ODE-System**

$\frac{d[pDNA - RNAII - RNAIunstable]}{dt}=-v_{1} + v_{2} + v_{3} - v_{4} - v_{10} + v_{11}-\mu\cdot\left[ pDNA-RNAII-{RNA}_{unstable} \right]$ *(2)*

$\frac{d[Rom]}{dt}= =-v_{1} + v_{2} + v_{13} + v_{20} - v_{23} - \mu\cdot[Rom]$ *(3)*

$\frac{d[pDNA - RNAII - RNAI - Rom]}{dt}= v_{1}- v_{2}- v_{13}- \mu\cdot[pDNA - RNAII - RNAI - Rom]$ *(4)*

$\frac{d\left[ pDNA - RNAII - s \right]}{dt}= -v_{3} + v_{4}- v_{5} + v_{9} - v_{15}- \mu\cdot[pDNA - RNAII - s]$ *(5)*

$\frac{d[RNAI]}{dt}= -v_{3} + v_{4}+ v_{18}- v_{21} - v_{25} - \mu\cdot[RNAI]$ *(6)*

$\frac{d[pDNA - RNAII - lo]}{dt}= v_{5}- v_{6}- v_{8}+ v_{16} - \mu\cdot[pDNA - RNAII - lo]$ *(7)*

$\frac{d[pDNA - RNAII - Primer]}{dt}= v_{6} - v_{7} - \mu\cdot[pDNA - RNAII - Primer$ *(8)*

$\frac{d[RNAII - lo]}{dt}=v_{7}+v_{8}-\mu\cdot[RNAII - lo]$ *(9)*

$\frac{d[pDNA]}{dt} = 2 \cdot v_{7}+v_{8} - v_{9} + v_{12} - v_{17} - \mu\cdot[pDNA]$ *(10)*

$\frac{d[pDNA - RNAII - RNAIstable]}{dt}= v_{10} - v_{11} - v_{12} + v_{13} - \mu\cdot[pDNA - RNAII - RNAIstable]$ *(11)*

$\frac{d[tRNA]}{dt}=-v_{14} - v_{15}+ v_{24}- v_{26}-\mu\cdot[tRNA]$ (12)

$\frac{d[RNAII]}{dt}= -v_{9} - v_{14} + v_{19}- v_{22} - v_{25} - \mu\cdot[RNAII]$ *(13)*

$\frac{d[tRNA - RNAII]}{dt}= v_{14} - v_{17} - \mu\cdot[tRNA - RNAII]$ *(14)*

$\frac{d[pDNA - RNAII - tRNA]}{dt}= v_{15} - v_{16} + v_{17} - \mu\cdot\left[ pDNA - RNAII - tRNA \right]$ *(15)*

$\frac{d[RNAI - RNAII]}{dt}= v_{25} - \mu\cdot[RNAI - RNAII]$ *(16)*

**Rate equations**

**Reaction 1:**

Building of a transient complex of plasmid bound RNAII, RNAI and Rom protein

*v*_1_ = *k*_1_ · [*pDNA* − *RNAII* − *RNAIunstable*] · [*Rom*] (17)

**Reaction 2:**

Breakup of a transient complex of plasmid bound RNAII, RNAI and Rom protein

*v*_2_ = *k*_2_ · [*pDNA* − *RNAII* − *RNAI* − *Rom*] (18)

**Reaction 3:**

Binding of RNAI to plasmid bound RNAII

*v*_3_ = *k*_3_ · [*pDNA* − *RNAII* − *s*] · [*RNAI*] (19)

**Reaction 4:**

Breakup of an unstable complex of plasmid bound RNAII, RNAI and Rom protein

*v*_4_ = *k*_4_ · [*pDNA* − *RNAII* − *RNAIunstable*] (20)

**Reaction 5:**

Elongation of the plasmid bound RNAII transcript

*v*_5_ = *k*_5_ · [*pDNA* − *RNAII* − *s*] (21)

**Reaction 6:**

Modification of the plasmid bound RNAII transcript

*v*_6_ = *k*_6_ · [*pDNA* − *RNAII* − *lo*] (22)

**Reaction 7:**

Plasmid replication

*v*_7_ = *k*_7_ · [*pDNA* − *RNAII* − *Primer*] (23)

**Reaction 8:**

Release of the elongated RNAII transcript

*v*_8_ = *k*_8_ · [*pDNA* − *RNAII* − *lo*] (24)

**Reaction 9:**

Hybridization of the RNAII transcript with the plasmid DNA

*v*_9_ = *k*_9_ · [*pDNA*] (25)

**Reaction 10:**

Conversion of an unstable complex of plasmid bound RNAII and RNAI into a stable complex

*v*_10_ = *k*_10_ · [*pDNA* − *RNAII* − *RNAIunstable*] (26)

**Reaction 11:**

Conversion of a stable complex of plasmid bound RNAII and RNAI into an unstable complex

*v*_11_ = *k*_11_ · [*pDNA* − *RNAII* − *RNAIstable*] (27)

**Reaction 12:**

Release of RNAI-RNAII complex from the plasmid DNA

*v*_12_ = *k*_12_ · [*pDNA* − *RNAII* − *RNAIstable*] (28)

**Reaction 13:**

Release of RNAI-RNAII complex from the plasmid DNA

*v*_13_ = *k*_13_ · [*pDNA* − *RNAII* − *RNAI* − *Rom*] (29)

**Reaction 14:**

Binding of RNAII to an uncharged tRNA

*v*_14_ = *k*_14_ · [*tRNA*] · [*RNAII*] (30)

**Reaction 15:**

Binding of an uncharged tRNA to plasmid bound RNAII transcript

*v*_15_ = *k*_15_ · [*tRNA*] · [*pDNA* − *RNAII* − *s*] (31)

**Reaction 16:**

Elongation of the plasmid bound RNAII transcript and release of the bound uncharged tRNA

*v*_16_ = *k*_16_ · [*pDNA* − *RNAII* − *tRNA*] (32)

**Reaction 17:**

Binding plasmid DNA to RNAII bound uncharged tRNA

*v*_17_ = *k*_17_ · [*tRNA* − *RNAII*] · [*pDNA*] (33)

**Reaction 18:**

RNAI synthesis

*v*_18_ = *k*_18_ · [*pDNA*] (34)

**Reaction 19:**

RNAII synthesis

*v*_19_ = *k*_19_ · [*pDNA*] (35)

**Reaction 20:**

Synthesis of Rom protein

*v*_20_ = *k*_20_ · [*pDNA*] (36)

**Reaction 21:**

RNAI degradation

*v*_21_ = *k*_21_ · [*RNAI*] (37)

**Reaction 22:**

RNAII degradation

*v*_22_ = *k*_22_ · [*RNAII*] (38)

**Reaction 23:**

Rom degradation

*v*_23_ = *k*_23_ · [*Rom*] (39)

**Reaction 24:**

Synthesis of uncharged tRNA

*v*_24_ = *k*_24_ (40)

**Reaction 25:**

Complex formation of RNAI and RNAII

*v*_25_ = *k*_25_ · [*RNAI*] · [*RNAII*] (41)

**Reaction 26:**

Degradation of uncharged tRNA

*v*_26_ = *k*_26_ · [*tRNA*] (42)
